# The makings of a modern epidemic: Endometriosis, gender, and politics

**DOI:** 10.1080/17441692.2014.986159

**Published:** 2014-12-12

**Authors:** Marci D. Cottingham

**Affiliations:** ^a^ Department of Social Medicine, University of North Carolina – Chapel Hill, Chapel Hill, NC, USA

Kate Seear's *The Makings of a Modern Epidemic* offers readers an in-depth critical examination of a modern illness from the perspective of the medical profession and the community of women who suffer its symptoms. She combines interview data alongside analysis of self-help and historical medical literature to tell the story of this contested illness and the dynamics of gender and discourse that it reveals. Estimated to affect 5–15% of all women, endometriosis involves the migration of menses tissue outside of the uterus causing severe cramping, bleeding and pain. Women may spend years suffering before receiving a diagnosis and treatment. More than just a case study of this gendered modern ailment, Seear provides an inspiring synthesis of constructionist theorising to reveal its foundational binaries. She examines the constructionist paradigm in contrast to realist and objectivist frames and explores the conceptual dimensions of risk, agency and the performative nature of disease. Methodologically, the analysis takes the form of a ‘pastiche’ of seemingly disconnected data points, unranked by hierarchy or narrative connection. While her analysis does not provide, nor claims to provide, a comprehensive overview of endometriosis, the portrait of the disease and the discourses surrounding its aetiology and symptomology cover a variety of sources making for a compelling analysis.

Physicians have struggled to understand the underlying mechanisms that lead to endometriosis. In the absence of clear facts and medical certainty, practitioners and patients alike turn to assumptions about women and the body. Historically, the disease has been thought to strike primarily ‘career women’ who delay having children, thus allowing menstrual tissue to build up as they experience a greater number of cycles throughout their lives. This classic victim-blaming approach leads to some disturbing ‘interventions’ – namely, the prescription of pregnancy as the solution to women's suffering regardless of economic or fertility status.

Beyond the discourse of the medical literature and the stories of women themselves, Seear turns to the surprisingly large self-help literature that has grown around the issue of endometriosis. Here, like her treatment of women's narratives, she questions the notion of empowerment. It is difficult to view the medical community's failure to diagnose or effectively treat the illness as a catalyst for empowering women. Self-help literature ‘performs’ endometriosis and the women who suffer it by portraying the disease as stable and surmountable. Women are individually advised to avoid the seemingly limitless substances in their environment that may contain harmful chemicals. Calls for political action that might compel government regulation of these numerous toxins are scant.

But medical experts and practitioners are not the only source of gendered discourse. Through technologies of the self, patients themselves call upon conflations of femininity with emotional instability and biological abnormality that gridlock them into the unfortunate position of renouncing the self as either delusional or incurable. Regardless of their resolve, the women in Seear's sample are forced to grapple with the always-lurking assumption that their illness is an illusion of the mind. Often told that the pain is in their head, women with endometriosis have to become rather demanding and headstrong if they are to convince physicians to take their symptoms seriously. This leads physicians to profile the ‘typical’ endometriosis patient as a defiant, headstrong woman. The patient–physician interaction itself constructs the portrait of a ‘typical’ patient, rather than this configuration of patient characteristics pre-existing the medical encounter.

Self-care – another concept that emerges in the analysis – refers to the myriad of practices that women engage in as they seek to ‘take charge’ of their health. When interventions such as surgery and drug therapies fail to provide some women with relief, they understandably turn to alternative treatments in the form of psychotherapy, acupuncture and other dietary and lifestyle modifications. Taking their treatment into their own hands may appear empowering, but Seear astutely questions the equation of self-care with empowerment. Underneath the notion of self-care lies the neoliberal assumption that the burden of care, even for the self, should fall back on individual women. To characterise this as ‘empowerment’ rings rather hollow.

In the way of weaknesses, Seear's subscription to a ‘pastiche’ method that suggests data remain only loosely connected, uncategorised by rank or narrative smacks of analytic timidity. Her account avoids this pitfall precisely because she does not allow data to ‘sit’ unanalysed in connection with the larger themes. While her openness to loose coherence and a plurality of views is laudable, the findings are most compelling when she situates her analysis against prior work in the study of endometriosis. Furthermore, it was unclear at times how her findings are unique to endometriosis medicine versus medicine overall. She states that an endometriosis epidemiologist ‘both assumes and reproduces “women with endometriosis” as a largely homogenous group, through seeking out patterns and connections regarding the *kinds of women* that are likely to develop the disease’ (p. 117, italics in original). This would seem to be the case for any disease in which physicians seek to identify ‘at-risk’ populations. Her analysis suggests that previous categorisation of patients emphasised the life choices of ‘career women’ over more physiologically based characteristics such as heavy menstrual flow. I wanted to better understand how these shifts took place – from emphasising the lifestyle of the ‘career woman’ to a focus on physiological ‘risk factors’ and back again to lifestyle characteristics implicated in diet and environmental factors. On the whole, Seear offers a compelling angle on this understudied topic. Her analysis of risk, the constitution of the patient through the medical encounter and the ambiguities of self-care all have important implications for the sociology of medicine and gender.

Marci D. Cottingham

*Department of Social Medicine, University of North Carolina – Chapel Hill, Chapel Hill, NC, USA*

*cottingham@unc.edu*

